# Wind driven semiconductor electricity generator with high direct current output based on a dynamic Schottky junction†

**DOI:** 10.1039/d1ra02308j

**Published:** 2021-05-26

**Authors:** Xutao Yu, Haonan Zheng, Yanghua Lu, Runjiang Shen, Yanfei Yan, Zhenzhen Hao, Yiwei Yang, Shisheng Lin

**Affiliations:** College of Microelectronics, College of Information Science and Electronic Engineering, Zhejiang University Hangzhou 310027 China shishenglin@zju.edu.cn; State Key Laboratory of Modern Optical Instrumentation, Zhejiang University Hangzhou 310027 China; Electric Power Research Institute of China Southern Power Grid Guangzhou Guangdong 510663 China

## Abstract

With the fast development of the internet of things (IoTs), distributed sensors are frequently used and small and portable power sources are highly demanded. However, current portable power sources such as lithium batteries have low capacity and need to be replaced or recharged frequently. A portable power source which can continuously generate electrical power *in situ* will be an ideal solution. Herein, we demonstrate a wind driven semiconductor electricity generator based on a dynamic Schottky junction, which can output a continuous direct current with an average value of 4.4 mA (with a maximum value of 8.4 mA) over 740 seconds. Compared with a previous metal/semiconductor generator, the output current is one thousand times higher. Furthermore, this wind driven generator has been used as a turn counter, due to its stable output, and also to drive a graphene ultraviolet photodetector, which shows a responsivity of 35.8 A W^−1^ under 365 nm ultraviolet light. Our research provides a feasible method to achieve wind power generation and power supply for distributed sensors in the future.

## Abstract 2

In this study, a generator based on a metal/semiconductor dynamic Schottky junction has achieved ultrahigh and continuous direct current output by harvesting wind energy.

## Graphical abstract

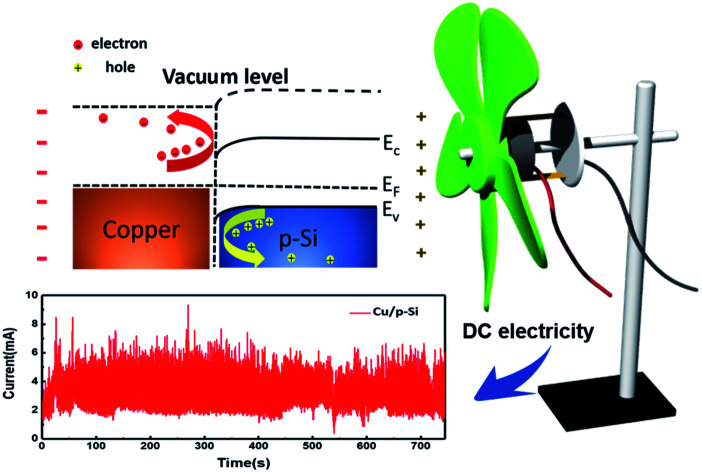

## 1 Introduction

With the development of the internet of things (IoTs),^1–3^ more and more sensors have emerged and been widely distributed, such as photodetectors,^4–6^ temperature sensors and vibration sensors,^7–10^ serving in the era of big data. For the rapid development of these scattered power-consuming sensors,^11,12^ a smart and *in situ* energy supply is in high demand for the smooth transmission of big data. Strikingly, energy storage devices have attracted more attention than the small and convenient electric generators.^13,14^ Nevertheless, lithium batteries need to be frequently recharged or replaced, which is very costly and time consuming.^15,16^ The best way of solving this problem is to create a small and convenient generator,^14,17,18^ which for instance makes use of the energy of low-frequency gentle breeze. It is hard to create a small and efficient generator, as clean energy sources like wind energy, tidal energy and geothermal energy can only be transformed into electricity by conventional large size equipment. For example, traditional wind turbines are very complex with a large number of blades, a transmission shaft, a gear case and a generator unit.^19,20^ What is more troublesome is that the conventional wind generator unit makes use of heavy coil rotation to cut the magnetic inductance line, and produces alternating current.^21–24^ In order to store energy and consume energy, the generator also needs an external rectifying circuit to change this into direct current. In contrast, direct current generators,^25–28^ like nanogenerators,^29–36^ which do not need an additional rectifying circuit to convert alternating current,^37–39^ have greater potential and will be more efficient to power sensors directly.^40,41^ However, for powering the distributed sensors in the IoTs and making the wind generator more convenient, there is an urgent need to invent a novel type of generator using a simpler device.

Herein, for the first time, we demonstrate a novel wind driven semiconductor electricity generator with ultrahigh direct current output, which is based on a dynamic metal/semiconductor van der Waals Schottky junction only. Our generator has an extremely high direct current output of 8.4 mA for a contact area of 0.45 cm^2^ between the metal and the semiconductor. Furthermore, this generator has been demonstrated to work continuously over 740 seconds with a stabilized output at an average value of 4.4 mA. As a proof of concept, using a special design with a polyimide insulating layer attached to the surface of a silicon rod, a portable turn counter has been obtained, whose mechanism is in contrast to the common magnetic turn-counting sensors.^42–44^ The wind driven semiconductor electricity generator has also been used to drive a graphene photodetector, which exhibits responsivity of over 35.8 A W^−1^. This demonstrates a potential method for direct power supply for driving widely distributed sensors in the age of the IoTs.

## 2 Experimental section

### 2.1 The device fabrication of the Cu/p-Si wind driven semiconductor electricity generator

The entire experimental setup was made up of a blade, n-type and p-type silicon rods (commercially purchased and prepared by the Czochralski method) and a copper sheet. A 55 mm diameter p-type silicon rod was cut into a thickness of 15 mm and washed with acetone, isopropanol and deionized water in sequence to clean the surface. After that, the native oxide layer of the silicon rod was dipped in hydrofluoric acid solution (purchased from Shanghai Aladdin Biochemical Technology with a CP of 40% and diluted to 10 wt%) for over 20 minutes, washed with deionized water and dried by nitrogen. After surface treatment, the surface silver electrode was prepared and formed a good ohmic contact with the silicon surface with low contact resistance by annealing in a high temperature furnace at 850 °C for 10 minutes. Due to the high temperature treatment (at 850 °C), in order to eliminate the influence of the surface oxide layer on the experiment as much as possible, it was necessary to remove the native oxide layer on the side of the silicon rod with diluted HF solution for 20 minutes. Then, the silicon rod with the prepared surface silver electrode was fixed to the blade to form the rotor structure of the windmill. As the stator structure of the windmill, the copper sheet’s surface was sequentially removed with ethanol and deionized water in order to complete the device fabrication.

### 2.2 Physical characterization methods

The current–voltage data were recorded using a Keithley 2400 and Agilent B1500A system. The data of the current and voltage over time were measured by a Keithley 2010 system with a sampling rate of 18 s^−1^. The rotation speed of the rotor structure and the contact area between surfaces were acquired by the image processing analysis software Image-Pro Plus (American MEDIA CYBERNETICS), where the rotation process of the generator is recorded by a video camera.

## 3. Results and discussion

**Fig. 1:**
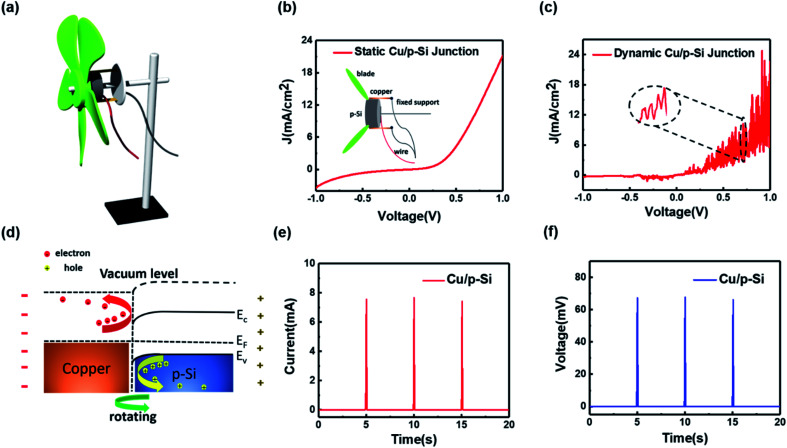
(a) Three-dimensional model of the wind driven semiconductor electricity generator. (b) *J*–*V* curve of the Cu/p-Si structured generator based on a static Schottky junction at bias voltages from −1.0 V to 1.0 V. The inset picture is the side view of the rotor structure and stator structure. (c) The rectification characteristic of the dynamic Schottky junction from −1.0 V to 1.0 V. (d) A schematic diagram of the internal carrier moving process between metal Cu and p-Si based on the dynamic Schottky junction and the signs on both sides indicate the outside potential difference. (e) The peak current output and (f) the peak voltage output for the dynamic Cu/p-Si wind driven semiconductor electricity generator. The contact area between the metal and the semiconductor is 0.4 cm^2^.

Fig. 1a illustrates the three-dimensional structure of the fabricated wind driven semiconductor electricity generator. The generator is composed of a windmill transmission blade, a semiconductor rotor structure and a metal stator structure, which are mainly supported by a metal bracket to form the complete device structure. It is worth noting that the metal and semiconductor are in close contact, as shown in the inset of Fig. 1b. When the copper sheet is attached with the p-type silicon rod in the static state, the static *J*–*V* curve of the formed Schottky diode can be measured, which is also shown in Fig. 1b. The rectified *J*–*V* curve, at bias voltages from −1.0 V to 1.0 V, demonstrates that a good static Schottky junction is formed between Cu and p-type silicon. In addition, the *J*–*V* curve of the dynamic Cu/p-Si Schottky junction at bias voltages from −1.0 V to 1.0 V is shown in the Fig. 1c. The wind energy collected is converted into kinetic energy, driving the semiconductor rotor structure rotating with the blade. Compared with the static *J*–*V* curve shown in Fig. 1b, it is pretty interesting to find that the dynamic process shows fluctuation of the *J*–*V* curve (magnified in the inset of Fig. 1c). Certainly, the *J*–*V* curve of the dynamic Cu/p-Si Schottky junction also exhibits the good rectification characteristic, which demonstrates the fine contact surface of the device. Similar to the *J*–*V* curves of conventional solar cells under light,^45–47^ the *J*–*V* curve of our generator in the dynamic process also deviates from the origin, which confirmed the existence of power generation (the partial enlarged view at the origin is shown in the ESI, Fig. S1†).

The physical mechanism of this generator can be explained by the dynamic establishment and destruction of the depletion layer, which causes the separation of diffused electrons and holes in the built-in electric field. When metals and semiconductors are in a relatively static state, the drift–diffusion equation and current density can be described as follows:

1 *J*_p_ = *J*_p|drf_ + *J*_p|dif_ = *epμ*_p_*E* − *eD*_p_∇*p*

2 *J*_n_ = *J*_n|drf_ + *J*_n|dif_ = *enμ*_n_*E* + *eD*_n_∇*n*

where *J*_p_, *J*_p|drf_ and *J*_p|dif_ are the hole current density, the hole drift current density and the hole diffusion current density, respectively, *J*_n_, *J*_n|drf_ and *J*_n|dif_ are the electron current density, electron drift current density and electron diffusion current density, respectively *μ*_p_ and *μ*_n_ are the hole and electron mobilities, respectively, *p* and *n* are the position-dependent hole density and electron density in the semiconductor, respectively, and *D*_p_ and *D*_n_ are the hole and electron diffusion coefficients, respectively. *E* is the built-in electric field and *e* is the elementary charge. In the schematic diagram in Fig. 1d, the p-type silicon rod and the copper sheet are in close contact and form the dynamic Schottky junction. The band structure of static Cu/p-Si is illustrated in Fig. S2 (ESI†). The work function of copper is about 4.48 eV,^48^ and that of p-Si is about 5.05 eV (the calculation is listed in Note S1, ESI†). So, when the copper contacts the p-Si surface, a built-in electric field is established between Cu and p-Si due to the differences in their work functions. At the same time, a depletion layer forms at the interface. When the p-type silicon rod driven by the windmill rotates against the copper sheet, the balance of the static Schottky junction between the interface is broken and a dynamic Schottky junction gradually forms. From eqn (1) and (2), the current density of the Cu/Si Schottky junction is composed of *J*_drf_ and *J*_dif_. We can assume that the contact area is unchanged during the whole rotation process, which means that the origin of power generation is when the balance of drift–diffusion is broken. Due to the continuous generation and destruction of the depletion layer, the otherwise diffused electrons and holes lose their paths for diffusing and are reflected or rebounded by the built-in electric field (the carriers’ movement processes are shown in Fig. 1d), which forms the direct current output. Therefore, the fluctuation of the *J*–*V* curve (Fig. 1c) during the dynamic process represents the output of power during the dynamic process. When the metal and silicon rotate relative to each other continuously, the generator outputs direct electrical current. The most unique feature in our wind driven semiconductor electricity generator is that it can output an ultrahigh direct current. In Fig. 1e and f, the peak current and voltage output are 7.6 mA and 67.1 mV, respectively.

Up to now, this output current of 7.6 mA is three orders of magnitude higher than the value reported for generators based on a moving Schottky diode.^37^ Due to the use of wind energy instead of the previous manual sliding, the pressure between the metal copper and the silicon rod has a better fit to generate a larger milliampere level direct current. Therefore, the wind driven generator with a Cu/p-Si structure based on a dynamic Schottky junction has been realized. As for our generator, it is more noteworthy that, owing to the output direct current being at the milliamp level and beyond the lower limit of the current analog value of 4 mA, it can also be used for the long-distance current transmission of analog signals.^49^

**Fig. 2:**
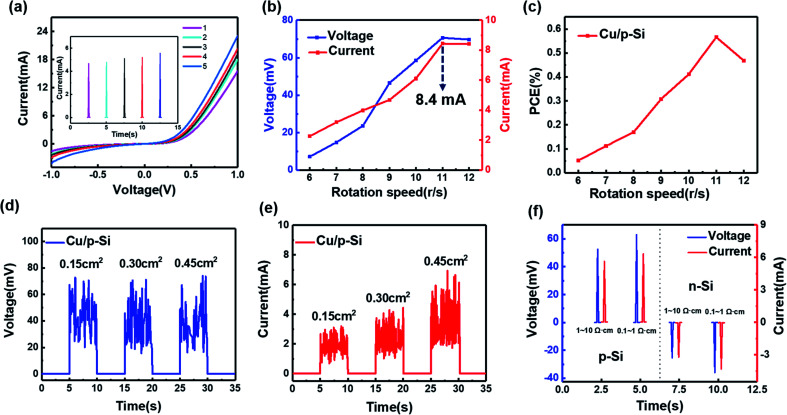
(a) The static *I*–*V* curves, where different colors indicate different leakage currents and the inset of the graph presents the corresponding output currents based on the Cu/p-Si structure (0.1–1 Ω cm). (b) The correlation of direct voltage and current output for different rotation speeds of the rotor structure. (c) The dependence of the PCE on the different rotation speeds. (d) The dependence of the continuous direct voltage output and (e) the continuous direct current output on the contact area at a rotation speed of 11 r s^−1^. (f) The output of the voltage and current with Cu/p-Si (0.1–1 and 1–10 Ω cm) and Cu/n-Si (0.1–1 and 1–10 Ω cm). The contact area is 0.4 cm^2^ at a rotation speed of 10 r s^−1^.

The output current under the dynamic situation needs to be further discussed. The output current is also related to the leakage current under the static *I*–*V* curve. As displayed in Fig. 2a, for the generator with the Cu/p-Si structure (0.1–1 Ω cm), the static *I*–*V* curve under different conditions has been measured (the surface of the silicon rod was polished with abrasive paper to achieve the different roughnesses), which represents the difference of the different leakage currents simultaneously. It is worth noting that the output current will increase gradually (the inset of Fig. 2a), when the leakage current increases. As mentioned in previous work, the output current has a positive correlation with the surface states.^25^ Also, there has been some recent research which has revealed this interesting phenomenon: the more leakage current under the static Schottky junction, the larger the output current will be.^38,39^ In order to explore the improvement of the generator performance, the output voltage and current of the generator *versus* the rotation speed has been presented in Fig. 2b. It can be seen that when the speed gradually increases, the output voltage has a certain positive correlation with the speed, as well as the output current. With the speed increasing from 6 r s^−1^ to 11 r s^−1^, the voltage increases from 7.2 mV to 70.7 mV and the current increases from 2.3 mA to the maximum value of 8.4 mA. The rotation speed increasing means that the built-in electric field is destroyed and established rapidly, while the voltage is limited due to the barrier height of the Cu/p-Si heterojunction simultaneously. Also, the current is limited by the drift–diffusion equation, where the root of the output current comes from the rebounding of the diffused carriers. So, when the blades’ rotation speed increases further, the output voltage and current of the entire generator gradually level off.

The power conversion efficiency (PCE) of the generator with the Cu/p-Si structure was also obtained. According to the energy conversion relationship, it can be described as follows:3
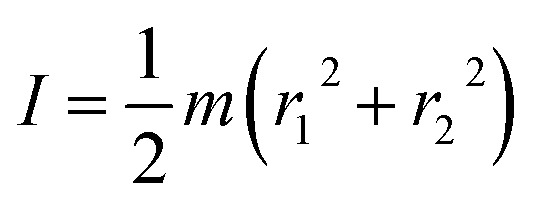
4
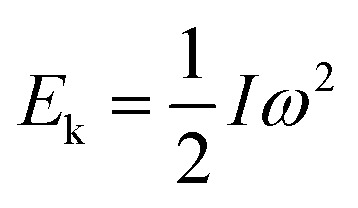
5
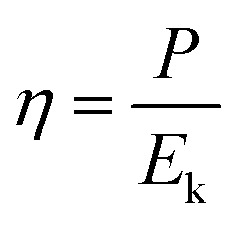
where *I* is the rotational inertia of the rotor structure, *m* is the quality of the rotor structure, *r*_1_ and *r*_2_ are the inner diameter and outer diameter, respectively, *E*_k_ is the kinetic energy of the rotor structure, *ω* is the rotational angular velocity, *P* is the output product of the output voltage and current and *η* is the PCE of the generator. Based on eqn (3)–(5), the PCE of the generator dictates the conversion efficiency of the kinetic energy of the rotor structure into electricity. In Fig. 2c, when the rotation speed was varied from 6 r s^−1^ to 11 r s^−1^, the PCE continued to increase with a maximum value of 0.56%. In addition to the influence of the rotation speed, the contact area between the metal and the semiconductor is also a major factor which influences the electricity output of a generator. In order to control the different contact areas between the metal and the semiconductor, different length of metal sheets have been designed for our generator. In Fig. 2d and e, the output voltage and current have been obtained with a fixed rotation speed of 11 r s^−1^. The contact areas are 0.15 cm^2^, 0.30 cm^2^ and 0.45 cm^2^. As shown in Fig. 2d, the values of the output voltage are similar and exceed 70.0 mV (the peak value). The fact that the output voltage depends on the materials’ work function difference but not on the contact area accurately reflects the mechanism of the generator. In Fig. 2e, the continuous current output is plotted for the different contact areas. The average value of the continuous output current increases from 2 mA to 4.7 mA with the increased contact area. From the mechanism of the generator, when the contact area of the metal and the semiconductor increases, more diffused electrons and holes crossing the depletion layer will be accelerated to rebound back to form a continuous current output in the dynamic Schottky junction. Moreover, the voltage and current output for different resistivities has been measured and are shown in Fig. 2f. The different resistivities represent different doping concentrations, which means the difference in work function. For the Cu/p-Si structure, the voltage changed from 52.5 mV to 63.0 mV and the current changed from 5.6 mA to 6.3 mA when the resistivity was changed from 0.1–1 to 1–10 Ω cm. For the Cu/n-Si structure, the voltage changed from −25.3 mV to −35.9 mV and the current changed from −3.2 mA to −4.3 mA when the resistivity changed from 1–10 to 0.1–1 Ω cm. So, the bigger the difference in work function between copper and the silicon rods, the more obvious the increase in the output voltage and current.

**Fig. 3:**
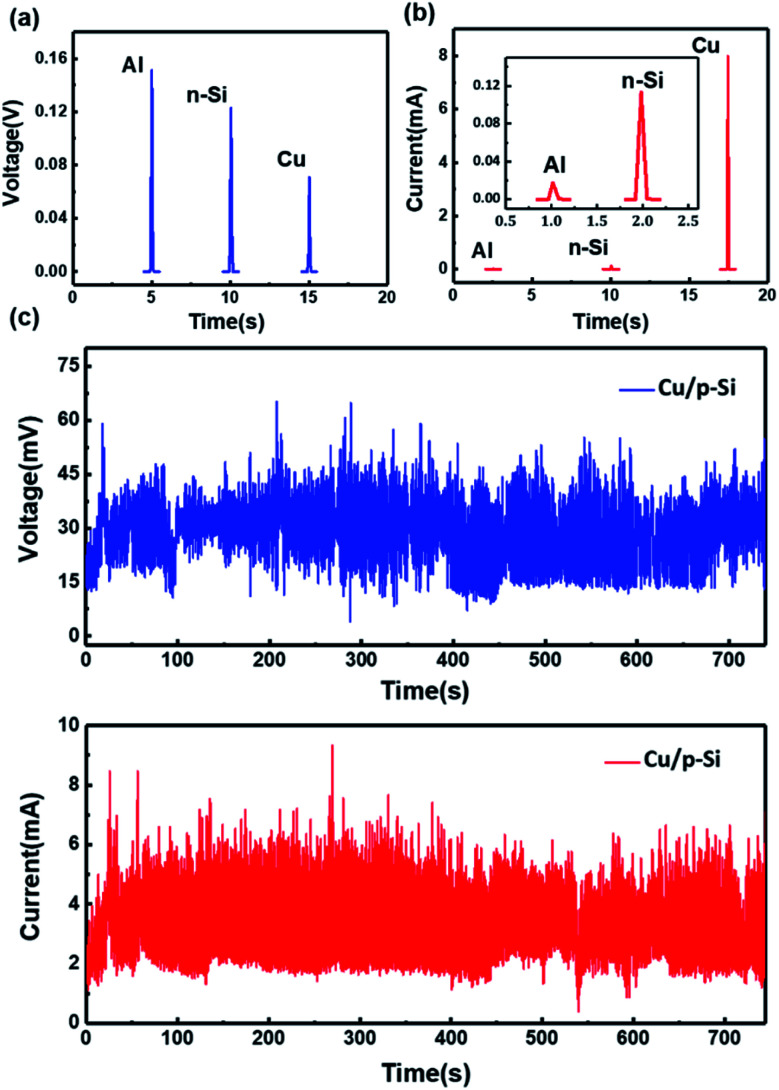
(a) Voltage output and (b) current output of the generators formed by using different materials in contact with p-Si. (c) The continuous direct voltage and the direct current output of the Cu/p-Si heterostructure-based wind driven generator over 740 s. For all, the contact area between the rotor structure and the stator structure is 0.45 cm^2^ and the rotation speed is 11 r s^−1^.

Furthermore, materials with different work functions have been used to test the direct electricity output, as shown in Fig. 3a and b. The work functions of aluminum and n-Si are 4.20 eV and 4.27 eV, respectively (the calculations are listed in Note S1 of the ESI†). Therefore, the work functions of these materials are smaller than that of p-Si, which causes the output electricity to be in the same direction due to the built-in electric field forming in the same direction. The peak of the voltage output has been shown for aluminum, n-Si and copper. For the aluminum/p-Si structure, a direct voltage output with an average value of 0.15 V has been achieved, shown in Fig. 3a. The result can also be repeated, as shown in Fig. S3 (ESI†). The output direct voltage of n-Si is 0.12 V, which is also shown in Fig. S4 (ESI†). Compared to copper, the difference between the work functions of p-Si and aluminum (or n-Si) are nearly 0.2 eV larger. Therefore, the generated voltages for Al and n-Si/p-Si are higher than that for Cu/p-Si on account of the differences in their work functions. In addition, the output currents are also measured and are shown in Fig. 3b. The peak of the output current of the Cu/p-Si structure is 8.0 mA. The peaks of the output currents of metal aluminum and n-Si are 17.1 μA and 118.0 μA, respectively (the comparison of multiple measurements is shown in Fig. S5 and S6 of the ESI†), which are shown in the inset in Fig. 3b. Overall, the generator with the Cu and p-Si structure shows better output characteristics based on a dynamic Schottky junction.

In order to explore the practical usage capability, the Cu/p-Si wind driven semiconductor electricity generator structure is fabricated. The continuous output voltage and current sustained for over 740 seconds has also been shown in Fig. 3c. With a rotation speed of 11 r s^−1^ and a contact area of 0.45 cm^2^, the continuous output voltage stabilizes at an average value of 40.1 mV and the continuous output current stabilizes at an average value of 4.4 mA. The stable output direct voltage and current have clearly demonstrated that our generator can continuously and effectively convert low-frequency wind energy into electric energy. At the same time, the static *I*–*V* curves before and after the experiment (Fig. S7, ESI†) show that there is almost no native oxide layer formed and no major damage to the surface of the contact area for the generator with the structure of Cu and a silicon rod, which shows its potential for practical applications. Furthermore, it is essential to prevent the interface from being oxidized ulteriorly, such as by covering the whole interface with an organic film,^50,51^ which is also worth exploring further.

**Fig. 4:**
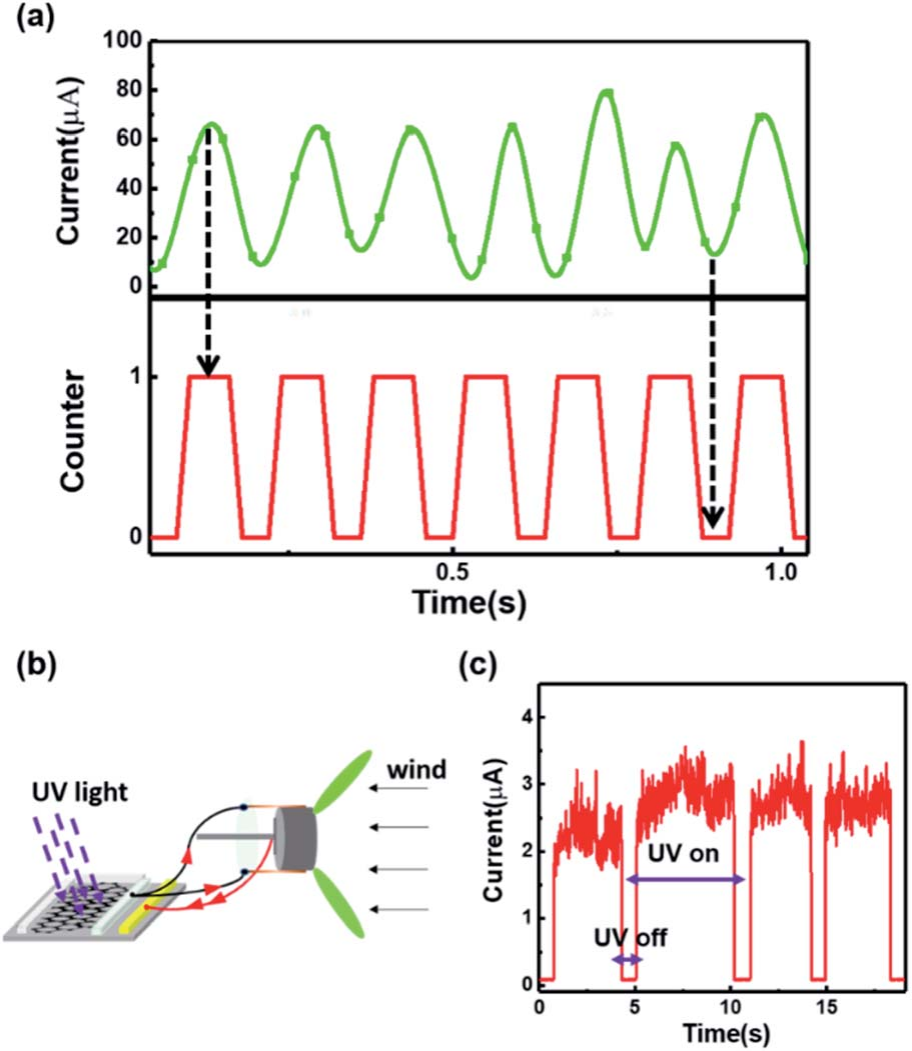
(a) Use as a turn counter to count the number of turns of the windmill. (b) Schematic of the generator driving a graphene/GaN ultraviolet photodetector under 365 nm ultraviolet light. (c) The photoelectric response of the graphene/GaN ultraviolet photodetector driven by our generator under 365 nm ultraviolet light.

In Fig. 4, examples of the practical application of the generator with the Cu/p-Si structure are presented. As shown in Fig. 4a, our generator can be used as a turn counter to record the number of turns. In order to achieve the target of counting, a kind of polyimide insulating layer is attached to the surface of the silicon, which can separate the metal sheets from the silicon rod surface. Sharp contrast can be achieved between the current peaks and valleys. The number of current peaks is in a good agreement with the number of turns of the generator, which demonstrates the realization of turn counting by our generator. Furthermore, a graphene/GaN ultraviolet photodetector has been driven by this generator, as shown in Fig. 4b and c. The graphene/GaN heterostructure device fabrication is given in Note S2 (ESI†). The two-dimensional structure diagram and a picture of the practical device are shown in Fig. S8 and S9 (ESI†), respectively. Under 365 nm ultraviolet light, the photodetector powered directly by this generator shows a responsivity of 35.8 A W^−1^ and worked steadily, which demonstrates the ability of our generator to provide a power supply for sensors.

## 4 Conclusions

In summary, a wind driven semiconductor electricity generator based on a dynamic Schottky junction has been demonstrated with a high direct current output. The novel mechanism is that the dynamic construction and destruction of the built-in electric field rebounded the diffusing carriers to form a continuous current output. The key important feature of the generator is that it is only constructed by the simple contact between a metal and a semiconductor, which can reduce the cost of present windmill power generation models through simplifying the power generation device and external conversion circuit. With the Cu/p-Si structure, continuous output current at an average value of 4.4 mA over 740 seconds is achieved. The application of the generator as a turn counter and power source for a graphene-based ultraviolet sensor is demonstrated successfully, which certifies the great potential of wind power generation to supply power for distributed sensors uninterruptedly.

## Author contributions

Xutao Yu: conceptualization, data curation, formal analysis, methodology, writing – original draft. Haonan Zheng: conceptualization, data curation, formal analysis, methodology, writing – original draft. Yanghua Lu: writing – review & editing. Runjiang Shen: help with experiments. Yanfei Yan: help with experiments. Zhenzhen Hao: help with experiments. Yiwei Yang: review & editing. Shisheng Lin: funding acquisition, resources, writing – review & editing.

## Conflicts of interest

There are no conflicts to declare.

## Supplementary Material

RA-011-D1RA02308J-s001
