# Supplementary material for: Wind driven semiconductor electricity generator with high direct current output based on a dynamic Schottky junction
Source: RSC Adv. 2021 May 26;11(31):19106–12. doi: 10.1039/d1ra02308j (PMC9033573; doi:10.1039/d1ra02308j)
Supplement: RA-011-D1RA02308J-s001 [file RA-011-D1RA02308J-s001.pdf]

Electronic Supplementary Material (ESI) for RSC Advances.
This journal is © The Royal Society of Chemistry 2021

Supporting information

Wind Driven Semiconductor Electricity Generator With High Direct Current Output Based On Dynamic Schottky Junction

Xutao Yu[a]†, Haonan Zheng[a]†, Yanghua Lu[a], Runjiang Shen[a], Yanfei Yan[a], Zhenzhen Hao[a], Yiwei Yang[c] and Shisheng Lin[a,b]*

[a]College of Microelectronics, College of Information Science and Electronic Engineering, Zhejiang University, Hangzhou, 310027, China

[b]State Key Laboratory of Modern Optical Instrumentation, Zhejiang University, Hangzhou, 310027, China

[c]Electric Power Research Institute of China Southern Power Grid, Guangzhou Guangdong 510663, China

[#]These authors contributed equally to this work.

*Author to whom correspondence should be addressed: shishenglin@zju.edu.cn

Note S1: The Fermi level of p-type Si and n-type Si

The Fermi level of p-type and n-type Si can be calculated by the formula below as below:

$$E_{F\text{-P}} \approx E_i - k_B T \ln \frac{N_A - N_D}{n_i} \tag{1}$$

$$E_{F\text{-N}} \approx E_i + k_B T \ln \frac{N_D - N_A}{n_i} \tag{2}$$

where $E_{F\text{-P}}$ and $E_{F\text{-N}}$ are the Fermi level of the p-type and n-type Si we used, E_i is the middle value of the band gap, k_B is the Boltzmann constant, T is the temperature, N_D is the electron concentration, N_A is the hole concentration, n_i is the intrinsic carrier concentration of Si which is about 1.5×10^{10} cm^{-3}. The hole concentration of the p-type Si rod we used is 1.30×10^{18} cm^{-3}. So, the Fermi level of the p-type Si is calculated about 5.05 eV according to equation (1). The electron concentration of the n-type Si we used is 4.63×10^{16} cm^{-3}. Therefore, the Fermi level of the n-type Si is calculated about 4.27 eV according to equation (2).

Note S2: The graphene/GaN heterostructure ultraviolet photodetector

The n-GaN had been obtained, which was grow on a sapphire substrate. The surface of GaN was cleaned utilizing acetone isopropanol and deionized water for 5 minutes respectively. Then the Ni/Au electrode was obtained by thermal evaporation on the front surface of GaN with a thickness of 70 nm. Monolayer graphene was grown on the surface of the copper foil through chemical vapor deposition (CVD) technique. The

monolayer graphene on a copper foil would be cover with polymethylmethacrylate (PMMA) on one side by spin-coating method. Heating some minutes to dryness it, the other side would be exposed to ultraviolet ray to break down the structure of graphene. All of copper foil would be put in home-made corrosive liquid ($CuSO_4$: HCl : H_2O=10g:50ml:50ml). Graphene would be transferred into deionized water to avoid the ions of solution attached. Then, graphene would be transferred to as-treated GaN substrate to form graphene/GaN heterostructure. After that, the silver electrode on the surface of graphene would be prepared and heated on the hot plate with the temperature 105℃.

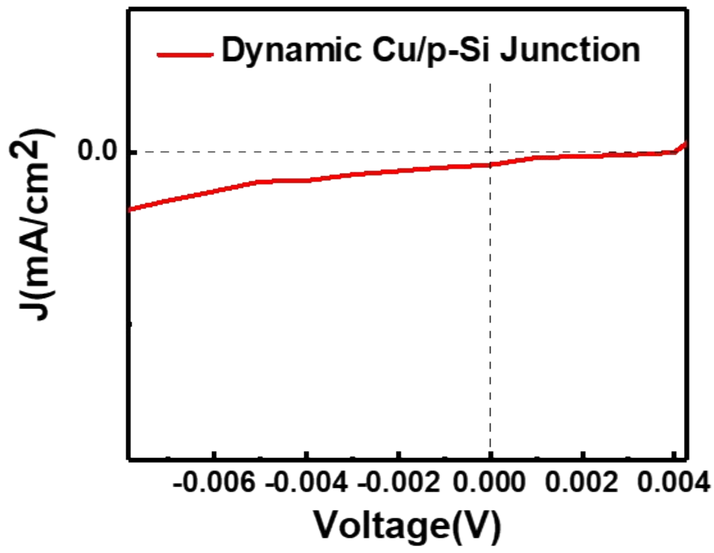

Figure S1: Adding two auxiliary lines to intersect at the origin, the dynamic Cu/p-Si junction deviate from the origin.

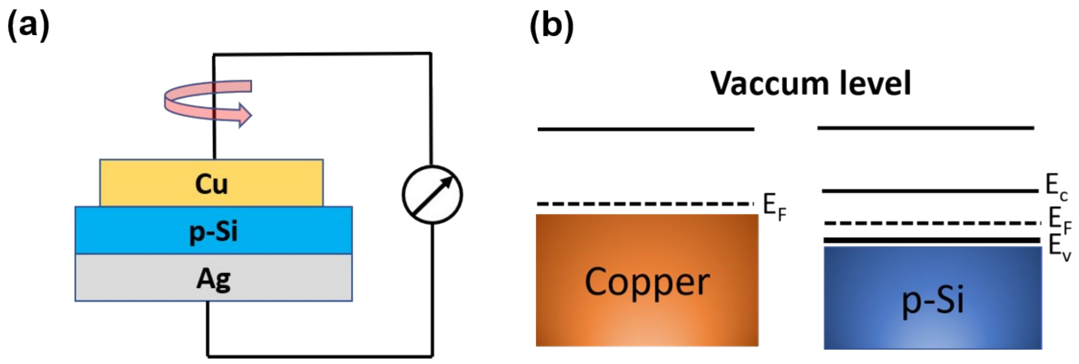

Figure S2: (a) The schematic diagram of Cu/p-Si wind driven semiconductor electricity generator. (b) The band structure of copper and p-Si relative to vacuum level before contacting to each other.

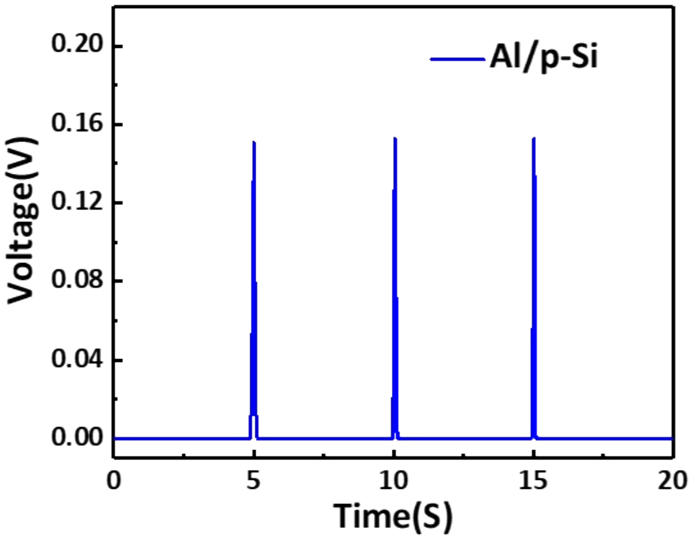

Figure S3: The output voltage of the structure of Al/p-Si wind driven semiconductor electricity generator. The contact area is 0.45 cm^2 and the rotate speed is 11 r/s.

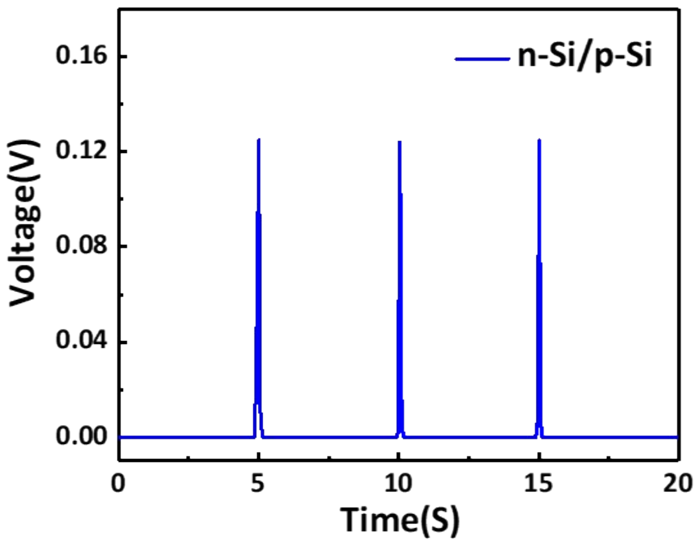

Figure S4: The output voltage of the structure of n-Si/p-Si wind driven semiconductor electricity generator. The contact area is 0.45 cm^2 and the rotate speed is 11 r/s.

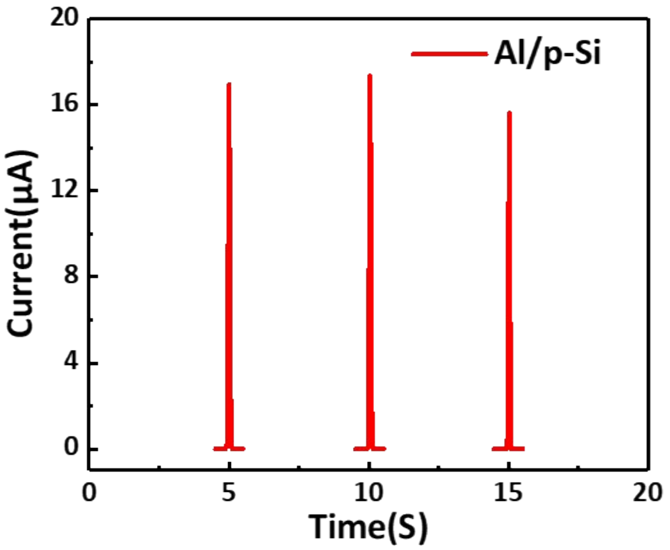

Figure S5: The output current of the structure of Al/p-Si wind driven semiconductor electricity generator. The contact area is 0.45 cm^2 and the rotate speed is 11 r/s.

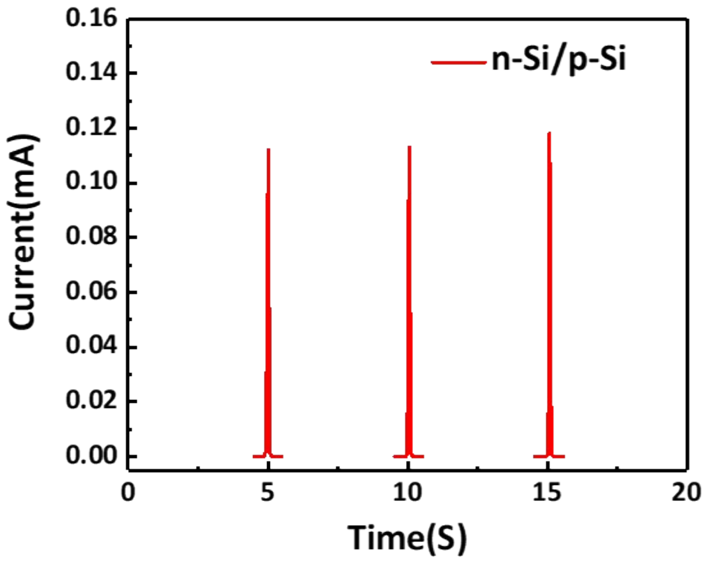

Figure S6: The output current of the structure of n-Si/p-Si wind driven semiconductor electricity generator. The contact area is 0.45 cm^2 and the rotate speed is 11 r/s.

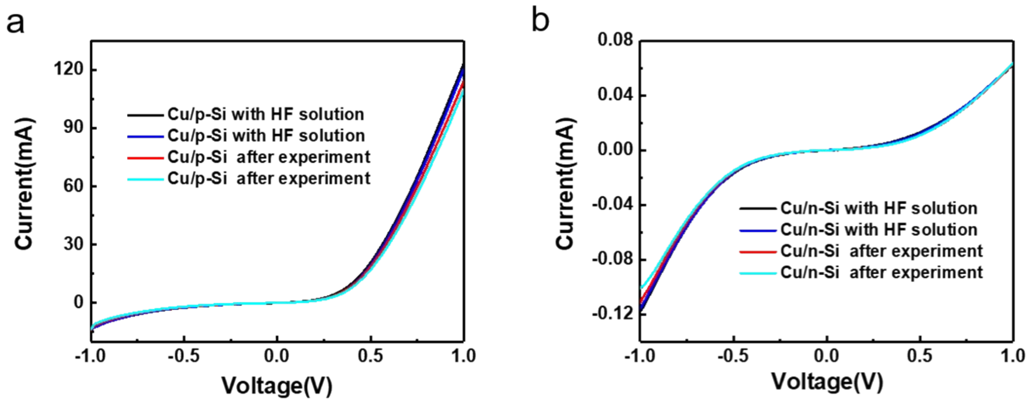

Figure S7: (a) The static IV curves of Cu/p-Si (1-10 Ω·cm) and (b) Cu/n-Si (1-10 Ω·cm): the black and blue curves are measured just after HF solution treatment, the red and cyan curves are measured after experiments.

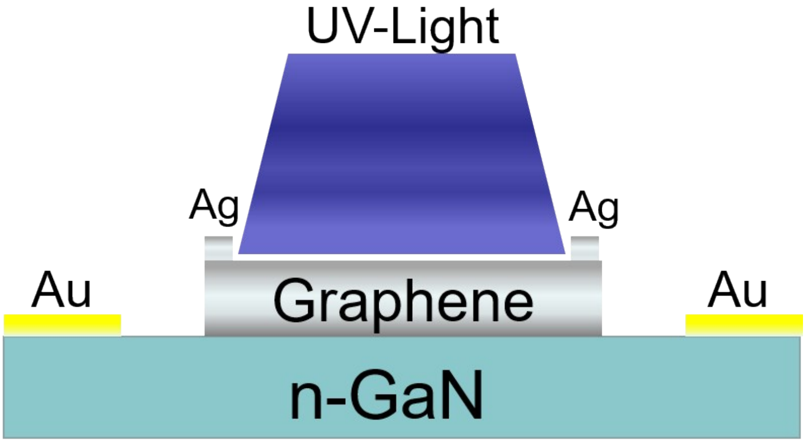

Figure S8: The diagram of two-dimension structure of Graphene/GaN ultraviolet photodetector under the ultraviolet light.

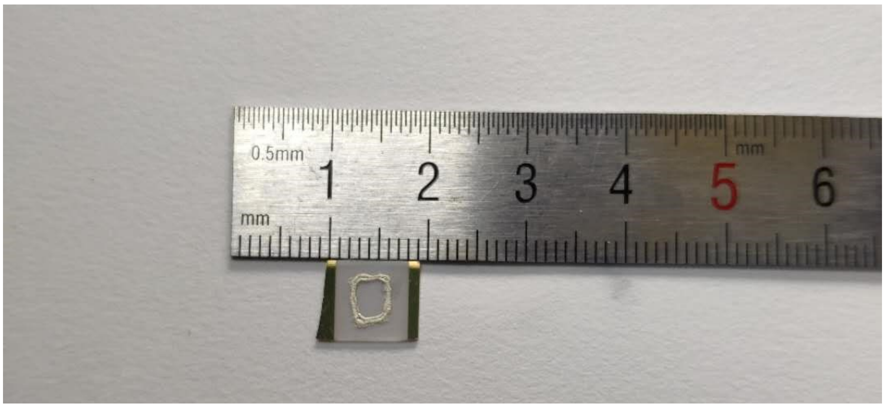

Figure S9: The physical picture of the structure Graphene/GaN ultraviolet photodetector, the effective UV-light (365 nm) absorption area is about 16 mm^2.
